# A review of the key issues associated with the commercialization of biobanks

**DOI:** 10.1093/jlb/lst004

**Published:** 2014-02-25

**Authors:** Timothy Caulfield, Sarah Burningham, Yann Joly, Zubin Master, Mahsa Shabani, Pascal Borry, Allan Becker, Michael Burgess, Kathryn Calder, Christine Critchley, Kelly Edwards, Stephanie M. Fullerton, Herbert Gottweis, Robyn Hyde-Lay, Judy Illes, Rosario Isasi, Kazuto Kato, Jane Kaye, Bartha Knoppers, John Lynch, Amy McGuire, Eric Meslin, Dianne Nicol, Kieran O’Doherty, Ubaka Ogbogu, Margaret Otlowski, Daryl Pullman, Nola Ries, Chris Scott, Malcolm Sears, Helen Wallace, Ma'n H. Zawati

## INTRODUCTION

Biobanks^1^ have emerged as a significant research tool, gaining support from both the scientific community and regional, national and international research funding agencies. However, developing and maintaining these platforms is expensive. Indeed, in a recent survey of operational personnel representing 456 biobanks, funding shortages concerned 71% of those surveyed and 37% identified ‘funding’ as ‘the biobank's greatest challenge.’^2^ Thus, unsurprisingly, biobanks may seek support from the private sector or philanthropic organizations, which have an interest in sustaining them as research resources.

The scientific community is also increasingly facing pressure to commercialize and translate their work, thus increasing expectations of industry partnerships.^3^ Funding agencies, in part, create and reinforce this commercialization pressure, by earmarking grants for projects that aim to bring products and therapies to the market within a short amount of time.^4^ This commercialization process creates a range of policy challenges for scientists, research participants, and funders.

The goal of this document is to outline the policy issues associated with the commercialization of biobanks and, where possible, to review the relevant evidence and/or ethical and legal norms.^5^ We do not attempt to provide suggestions for policy reform. Nevertheless, given that securing funding for biobanks remains a challenge and that researchers are increasingly pressured to work with industry and to rapidly translate their work, a scoping document of this nature—one that draws together international expertise and relevant research—seems timely and, we hope, can stand as a resource for future research and policy work.

‘Commercialization’ can refer to a number of different activities. It can refer to the commercialization of biobank resources (data or samples of human biological material) or of research results derived or products developed from those resources. It can also refer to publicly funded biobanks partnering with or receiving funding from private, for-profit entities like biotech companies, pharmaceutical corporations, or the medical device industry. For the purposes of this scoping document, we will largely focus on the introduction of private funding and partnerships to an existing, publicly funded biobank. This focus is justified because many biobanks are not-for-profit entities associated with larger (often publicly-funded) bodies, such as universities and hospitals, and funded, often through short-term grants, by these bodies, by the government, or by a mixture of public and private funds.^6^ Survey evidence indicates that these biobanks are greatly concerned with securing adequate long-term funding.^7^ As Meijer and colleagues note, with the expansion and change in the size, goals and structure of biobanks, ‘stable sources of core funding will be necessary in most cases, from the public sector, patient organizations and private foundations.’^8^ Given this reality, we believe that commercialization will particularly and uniquely impact publicly supported biobanks, as they consider various means, including private partnerships, to ensure financial security.^9^

Of course, biobanks vary greatly in organization, priorities, and funding,^10^ and thus commercialization will affect different biobanks in different ways. Not every biobank will seek commercial partnerships in response to financial pressures, and, indeed, delineating which factors (eg type of biobank; organizational structure; research goals and focus) influence the development of private partnerships may be an important question for future research. Indeed, we hope this overview of issues will inform these future funding decisions.

In addition to our primary focus on the introduction of private financing and partnerships, some of the emerging research and commentaries on the issues associated with commercialization are relevant to a range of other activities and are worth considering here. For example, issues of public trust can be triggered by a variety of commercialization practices, as we will see. Thus, where appropriate, we will discuss commercialization activities beyond just the introduction of private funding.

Before turning to the issues at hand, it may be useful to set out some of the scenarios in which commercialization may occur. In this paper, as noted, we largely concentrate on the introduction of private funding to a pre-existing publicly funded biobank. In such a case, a chief concern for the biobank will be retaining participants who were, presumably, originally recruited with an understanding that the biobank would be a ‘public good.’ In this case, issues related to consent, participant retention, and withdrawal of biological material and associated data are particularly important. We can contrast this with a scenario in which the biobank is a public-private partnership from its inception. In such as case, enrollment of participants and obtaining consent to commercial imperatives, if any, are likely to be the prime focuses of the biobank. The different situations of these biobanks may inform and nuance the discussion that follows.

### ISSUES

#### Public Trust and Public Expectations

Trust is critical in determining whether people will participate in and support biobanking research. Public trust and how it influences the public's support for science is complex and influenced by many factors, including perceptions of the actors and institutions involved in research.^11^ There is little doubt that the general public places a good deal of trust in the university-based scientific community. For example, a 2013 survey of United Kingdom residents commissioned by the Wellcome Trust found that 66% percent of respondents completely trusted or had a great deal of trust in university scientists.^12^ However, it also showed that trust evaporated quickly if scientists worked for either industry (trusted by 32% of respondents) or government (trusted by 34% of respondents).^13^ This has also been the case in Australia, where the Swinburne National Technology and Society Monitor has consistently found high public trust in scientists, universities and government research organizations.^14^ Trust in public research institutions remains high even for controversial medical research (including some research in genetics), but decreases significantly with industry involvement.^15^

The involvement of industry in research seems to have considerable influence on public trust in science, irrespective of the type of research.^16^ This holds true in the biobanking context, as well. Several studies show that many participants have a negative attitude towards the involvement of commercial entities in biobanks and biobanking research.^17^ A survey of 1,201 Albertans found that public trust in biobanking research decreases substantially if industry is involved: 45.1% of those surveyed indicated they had ‘a great deal’ of trust in university-funded scientists; 19.5% had ‘a great deal’ of trust in university scientists funded by industry; and only 6% had similar trust in biobanking research conducted by for-profit industry.^18^ A recent public opinion study of cancer patients similarly showed that 40.4% of participants reported having ‘a great deal’ of trust in biobanking research performed by government-funded university researchers as opposed to 16.3% and 3.2% of respondents reporting ‘a great deal’ of trust in industry-funded university research and research conducted by industry, respectively.^19^ Interestingly, a study by Critchley and Nicol suggests that the type of organization (eg private entity versus university) has a greater impact on public trust than funding source (eg private versus public funding), though the source of funding also influences public trust.^20^ Though their study was not specific to biobanking research, it may suggest that public biobanks which receive private funding will be better able to maintain public trust than those that are completely privately owned.

Many factors may account for a negative view of private investment in public biobanks. For instance, there is some evidence that members of the public fear that their donated biological samples and associated health data will be used in ways they find morally problematic, such as in research that may be stigmatizing or discriminatory to them and/or their communities^21^ (for example, research connecting mental health with racial or ethnic background). Some biobank participants fear losing control over how their samples and data are used and with whom they are shared, and this fear may be heightened when for-profit companies invest in publicly funded biobanks. This notion is manifested in recent research by Critchley, Nicol, Otlowski and Chalmers (unpublished manuscript), which found that concern about sharing of genetic data with private entities was the chief reason for the significant erosion of support when genetic tests were conducted by a private company compared to those controlled by the public health care system.^22^

The public generally supports biobanking research,^23^ though the terms on which people will participate and the expectations they have regarding participation are not uniform and differ on key matters like benefit sharing.^24^ Some people participate for altruistic reasons, without expecting any benefits in return.^25^ Other people are more likely to participate in biobanking research if they believe that biobanks will produce beneficial results for future patients and society.^26^ Some participants do expect to receive something in return, including monetary reward, reduced costs of the (potential) clinical benefits derived from biobanking research, or the return of research results.^27^ These observations also seem connected with the idea that private interests, including the introduction of industry funding, may limit sharing of biological material and associated data, prevent results from being returned, or limit public access to health benefits derived from private or proprietary research. The belief that public access to benefits will be reduced has been found to be an important reason for the decrease in public support for industry-based medical research (relative to publicly funded researchers).^28^ This finding, combined with the public's desire for biobanks to share results and advance health care, suggests that some sort of benefit sharing arrangement or return of benefits back to the community may help the public accept the commercialization imperative.^29^ Involvement of commercial entities, in the form of financial support for public biobanks, thus has the potential to change the motivations and expectations of the general public, hence altering the relationship between participants and biobanks. Of course, the particular way in which financial support from industry will affect this relationship and related motivations and expectations depends on many factors and may differ between distinct groups within the public.

The hype from genomics generally redounds to biobanks and this too might impact public expectations, such as the belief that biobanks will result in benefits in the near future.^30^ If the benefits of biobanking are seen to be oversold, the involvement of commercial entities in the form of financial support is likely to garner even greater suspicion.^31^ The expectation that public investment should yield results for the public good can be used to justify the creation of publicly funded biobanks and also the adoption of a range of biobanking policies that may be ethically or legally contentious (eg the use of a general or broad consent approach).^32^ The involvement of industry can create questions about the degree to which the biobank research is being done solely—or even primarily—for the public good, thus compromising, in the eyes of the public, the validity of the justification for the adoption of these policies.

It seems reasonable to think that if the public perceives scientists from private research organizations to be more self-interested (ie for profit) than interested in the public good, this could diminish trust in biobanks that previously involved only publicly funded scientists, and thus hinder public participation. Yet this simple calculus has several limits. First, a sweeping statement that all public institutions are more trustworthy and private ones have motives contrary to the public good is a glaring oversimplification. Although we have referred to research which suggests that government-funded scientists are perceived to be more trustworthy than those working in private industry, there is an underlying distrust of the government as an overseer of biobanking practices.^33^

Second, trust in actors and institutions differs between various groups and may be influenced by factors such as race, gender, education, political or other ideology, religion, geography, and whether the individuals in question are impacted by a disease.^34^ However, some evidence suggests that demographic factors have little impact on biobanking preferences and should not be viewed as determinative of peoples’ biobanking preferences.^35^

Third, not everyone will reject participating in a biobank that has some commercial elements. Studies suggest that patient groups tend to be more accepting of pharmaceutical involvement than other civic groups, for example, and participants may be more at ease with commercialization elements if it means the biobank is ‘governed or regulated’ appropriately.^36^ Additionally, not all ‘private’ actors are seen identically and not all ‘public’ sources are deemed trustworthy. The actual governance and operations of the biobank, whether it is partly or fully privately (ie pharmaceutical, philanthro-capital) or publicly (ie government, non-governmental, or government-legal) funded, and the nature and goals of the biobank likely influence individual perceptions of trust.^37^

Although considerable efforts have been made to understand the attitudes of the general public, patients, and other groups, and assess their willingness to participate in biobanking initiatives, more research is needed especially as it relates to the involvement of commercial entities in biobanking, in the form of financial support or access by privately-funded scientists to biobank resources.^38^ Future research could focus on determining the perceived aspects of commercial involvement in biobanking that affect public trust (eg losing control over samples, misuse of biological samples and data, limited access to the benefits of research, the return of results, increased secrecy and limited sharing, and research misconduct and undertaking unethical research).^39^ Participants may lack knowledge about biobanking and commercialization and desire to receive further information. If public communication and engagement are provided, will this lead to greater acceptance of commercial involvement in publicly-funded biobanks? And if so, under which conditions will the general public accept various types of commercial involvement in publicly funded biobanks? Further research could also explore whether and how the introduction of private funding or partnerships impacts the weighing of values and preferences that factor into an individual's decision about whether to participate in biobanking research.^40^ By identifying how public trust is affected by different commercialization practices, future scholarship can simultaneously focus on the related issue of how to build or increase trust in public biobanks that partner with private entities. It may be, for example, that certain approaches to public-private partnerships—such as arrangements for benefit sharing—or actions by private entities—such as providing transparent information about intentions and goals and implementing open communication policies—will minimize the erosion of public trust that may accompany commercialization.

The current lack of consensus on many key biobanking issues^41^ combined with strong public reactions to recent controversies relevant to biobanking, albeit in a different context (eg *Havasupai Tribe versus Arizona Board of Regents*; destruction of 5 million blood samples by Texas Department of State Health Services), should remind us that trust can easily sway and may have a profound impact on biobanking endeavors.^42^

#### Conflicts of Interest

Commercialization may result in tension between the interests of private partners and those of the researchers or organizers of the biobank, which raises a number of interrelated concerns. If commercial entities contribute funding for biobanking research, scientists ‘may relinquish part of their independence over the development of the research.’^43^ Or procuring private investment or promoting the interests of commercial partners may trump other interests or priorities.^44^ For example, a biobank might authorize secondary uses of data or samples that either risk misuse of the resource or are inconsistent with the consent of participants in order to secure private funding. Likewise, scientists may replace basic, essential research with commercially-profitable research.^45^ Similarly, commercialization in the form of financial relationships between private entities and university scientists could compromise research integrity.^46^ For example, studies in other fields of biomedical research have shown that ‘industry-sponsored research tends to draw pro-industry conclusions.’^47^ Public perception of biomedical science as a tool for the ‘public good’ may be negatively impacted by these outcomes.^48^ As Hoeyer observes, ‘commercial incentives might work contrary to the ambition of using stored tissue to address public health problems, both by impeding some research collaborations and by modifying the research agenda and our shared understanding as to what constitutes an important health problem.’^49^

#### Consent

The issue of consent has proven to be one of the most divisive topics in the context of biobanking research. High profile tissue banking and genomic research cases—albeit not involving publicly funded population biobanks—and subsequent media attention surrounding these cases highlight the sensitivity of the consent issue and may contribute to increased scrutiny of biobanking practices.^50^

Consent issues typically arise in connection with the down-stream commercialization of products and therapies derived from research on biobank samples and data. Informed consent laws and norms require that researchers inform participants about potential commercial uses of their biological samples and data. But consent issues also emerge when considering the introduction of financial support by private entities to public biobanks, particularly if this possibility had not been addressed in the initial consent.

The importance of informing research participants about potential commercial applications resulting from research on their samples and data—an inevitable byproduct of industry involvement—has been endorsed by numerous guidelines and recommendations.^51^ Disclosure is required, in part, because knowledge of potential commercial uses has been shown to be a relevant factor in the decision of individuals to participate in biomedical research. As noted above, studies^52^ have reported that participants may see commercial applications at odds with their initial reasons for and expectations regarding participation.^53^ It is an ethical imperative that scientists be transparent regarding intended commercial use and address participants’ concerns related to commercialization.^54^ Again, depending on the nature of the commercialization agreements, these kinds of concerns may be a natural consequence of industry funding of a public biobank.

Given the increasingly common use of ‘broad consent’^55^ by biobanks, there may be questions about the sufficiency of consent if commercialization plans and goals are not clarified at the outset. Many ‘broad consents’ forms in current use may refer, with legally sufficient specificity, to possible future commercial applications or industry involvement. However, if commercialization issues are not addressed, engaging a private entity post-original consent may necessitate a re-consent process.^56^ In addition, an overly broad or generic reference to future commercialization activities may be insufficient. It has been argued, for example, that obtaining a broad consent to use samples for a wide range of commercial and non-commercial research in the future may be legally problematic and may require the provision of more detailed information about the nature of the commercial activity.^57^

Although informed consent is a mechanism for protecting participants’ interests and promoting their autonomy, it has limitations.^58^ Consent procedures need to be coupled with other governance mechanisms, such as ongoing monitoring by oversight committees, in order to ensure that biobank resources will be put to the best possible uses.^59^ Prior consultation and communication with communities and interest groups should also be considered.^60^ As Luther and Lemmens note, ‘an over-emphasis on consent, without sufficient attention to societal, economic, structural and other challenges to individual decision-making, may undermine rather than protect the interest of individuals’.^61^ As such, any consent mechanism or process that is put in place to address the involvement of private entities should be enhanced by appropriate, long-term oversight.

#### Ownership, Access, and Control

The law relating to ownership and control of human biological material varies in different countries, and, in some countries, remains unclear.^62^ In the United Kingdom, while courts have accepted that donors have some property rights in tissue set aside for future use, this right does not extend to tissue collected for research purposes.^63^ In the United States, courts have consistently held that individuals do not retain ownership of material donated for research.^64^ However, individuals may still exercise some degree of control over biological materials, as they are able to withdraw from research.^65^ And the existing jurisprudence may be limited by the facts, making its application to other situations uncertain.^66^ As a result, the law regarding ownership and control remains unclear.^67^ In Canada, while courts have not decided the issue of ownership of human biological material used in research, individuals have a common law right to access and, likely, control their health information.^68^ In Estonia, donated samples are owned by the institution overseeing the biobank.^69^ In Portugal, the participant owns the donated samples.^70^ Iceland does not use the term ‘ownership’ in its treatment of research samples.^71^

Additionally, survey research tells us that there is no consensus within the general public regarding who owns human biological material taken, stored and used for research. For instance, a survey of 1,201 Albertans revealed that 44.3% believed that the institution owned the samples, 25.7% felt that those who donated the samples owned them, 23.1% felt that the scientists owned the samples and 6.9% believed that the research funder owned the samples.^72^

As a result of this diversity of positions and legal uncertainty in several jurisdictions, many scholars have called for clarification on the proprietary rights (eg access, control) of donors, biobanks and researchers.^73^ The involvement of private entities in a public biobank, and a concomitant need to understand who controls samples, seems likely to make clarification even more imperative.

#### Sustainability and Bankruptcy

Biobanks are expensive to maintain.^74^ A lack of sufficient funding may have a dramatic impact on sustainability and raise challenging policy issues. Two recent bankruptcies of private population biobanks, Genizon in Québec, Canada and deCODE Genetics in Iceland, illustrate the ethical and policy dimensions of the issues raised in this context. Both biobanks were privately owned and recruited their participants from homogeneous population groups originating in geographically determined locations.^75^

Given these similarities, it is interesting to note the different outcomes of bankruptcy in the two cases. deCODE (with 140,000 samples from the Icelandic population) first declared bankruptcy in 2009, selling its assets (including its biobank) to Saga Investments LCC. Saga is a group comprising some of the original investors in deCODE who kept running the company with a similar management structure. Following an unsuccessful attempt to pierce the direct-to-consumer (DTC) market, the company was acquired by the biotechnology giant Amgen for US$415 million.^76^ So far, the sale has not affected the management and objectives of the biobank, which remains located in Iceland.^77^ As part of the deCODE licence, the government required that the biobank stay in Iceland, even if third parties became involved. However, Amgen has not offered any written guarantee that this arrangement will prevail over time and no court decision has been rendered to that effect. Genizon (with 50,000 samples from the French Québec founder population) was a Québec-based private biotechnology company in the business of mapping and identifying genes for complex disorders. It filed for bankruptcy and liquidated its tangible assets, excluding the biobank, in 2011.^78^ Later that year, the Superior Court of Québec mandated Génome Québec (GQ), a provincial public funding agency, to act as the trustee of the biobank. In late 2012, Genome Québec launched a call for tenders with the hope of transferring Genizon's biobank to a Québec research centre.^79^ Following the closure of the call for tenders, Genome Quebec turned down two valid bids by public groups deciding instead that it would continue managing the Genizon biobank by itself.^80^

In cases like this, what should happen to the samples and data of the participants? Can they be sold like other types of material assets? Transferred to another country? Or should they be destroyed instead? Responses to these questions could have far reaching implications for participants’ privacy, autonomy and dignity. In the case of bankruptcy, the biobank's policies, especially those regarding data privacy and data sharing, are relevant for courts to determine the extent of what can be done with the participants’ data and samples. The informed consent form signed by the participants will also play an important role in that respect, by creating conditions that should be respected post-bankruptcy. In this type of bankruptcy cases, US experts have alluded to the possibility of legally appointing a Consumer Privacy Ombudsman (CPO) to assist the court on privacy aspects of the process.^81^ However, to require this special procedure, the proposed liquidation should meet strict criteria that likely would not apply to most cases involving genomic biobanks.^82^ Given the sensitive nature of medical data, including genetic data and tissue samples, and the importance of preserving public trust in science, it appears that clear policies are required. Those policies should give weight to the promises made to research participants at the inception of a biobank and ensure more predictable outcomes in case of bankruptcy. However, developing such policies may be challenging, as issues relating to bankruptcy or biobank closure are rarely priorities at the inception of a biobank project^83^ and tend to be considered only in times of hardship.

#### Access Agreements

To protect the professional interests of scientists and the rights of research participants, biobanks are increasingly making use of a variety of access agreements. Usually some of the data from the biobank (eg summary reads, genotype frequencies, a limited amount of de-identified clinical data annotation fields) will be made available openly while the more sensitive data (eg gene expression data, raw genotypes, specific demographic data) and samples, if accessible at all, will be made available only to researchers who agree to comply with the terms of these access agreements.^84^ This second mode of access is usually referred to as ‘controlled access’ or ‘managed access’ by the research community. A biobank access committee will usually have the responsibility of reviewing these access agreements and granting access to approved users. An intrinsic limitation of any current access agreement is the limited capacity of biobanks to effectively monitor compliance and sanction potential infringers.^85^

Access agreements offer important advantages to biobanks but can also generate substantial issues which the biobanks should strive to minimize. The current speed of progress in informatics and bioinformatics has made it increasingly possible to re-identify individuals from a small amount of genetic and/or clinical data and a matching database, limiting the effectiveness of traditional means of protecting the identity of research participants in open biobank projects (eg anonymization, coding, and de-identification).^86^ Given this reality, it seems prudent to add another layer of protection that does not solely rely on technology.

However, it should be recognized that the imposition of an access agreement severely limits the open science nature of a biobank. Even the simplest access agreement containing a minimal number of restrictions may deter a substantial number of scientists from applying.^87^ Similarly, agreements that restrict access to or dissemination of research data may undermine the free flow of scientific knowledge. An important related issue is the risk that some biobanks, in an attempt to meet their obligations to protect the rights of participants, will impose overly restrictive or unacceptable conditions on members of the scientific community. Clauses requiring users to sign collaboration agreements with biobanks, to agree to overly long moratorium periods before using the data, to return enriched data to the biobank, or to give a first right of refusal on potential emerging intellectual property are all examples of provisions that could severely limit the open science nature of a biobank. Requiring an unacceptably high amount of money as compensation for the provision of samples (under the pretense of cost recovery) would have a similar effect and could also be considered a commercial sale of the human samples, which is illegal in many jurisdictions.^88^ This potential for misusing access agreements should be recognized^89^ and biobank administrators should be careful to use these agreements in a way that will only minimally restrict open science for the real benefit of research participants. Therefore, the publication of consensus statements or harmonized models of acceptable clauses for access agreements might have a very positive impact on the current practices of biobanks.^90^ However, in some cases, uniform policies may not address the unique circumstances of a particular biobank and thus context-specific clauses will be preferable.

## CONCLUSION

In addition to the issues examined above, introducing private funding into previously publicly-funded biobanks may exacerbate a range of other ethical issues.^91^ For example, how to recognize the right to withdraw from research in the context of biobanking is a controversial matter.^92^ The introduction of private entities or funding may further complicate the withdrawal issue because it may create obligations on scientists to share data with certain parties, including private partners, and data disseminated to them may be impossible to recall if a participant withdraws from the study.^93^ Similarly, the involvement of private funders may exacerbate privacy issues or increase tensions related to privacy. While funding itself is likely not an issue *per se*, if biological material and data are shared with, for example, pharmaceutical companies, participants may feel as though their privacy has been violated, which, as discussed above, may negatively impact public trust in biobanks.^94^ Finally, the proliferation of biobanks around the world and other technological innovations are increasingly making possible wide-spread sharing of data and biological samples, a practice which is encouraged in order to realize the economic and scientific potential of biobanks.^95^ This practice may be impacted or hindered by the introduction of private funding and collaboration with private entities, as the expectations of private entities and agreements governing such partnerships may create sharing barriers. Efforts to develop harmonized policies and interoperability between biobanks may address some of the issues that arise in this regard.^96^

Because biobanks are costly to develop and maintain, biobank developers are looking to private investment to facilitate the long-term sustainability of these important research tools. In addition, increased pressures to commercialize—now ubiquitous in the policies of public funding agencies and universities—have encouraged biobanks to partner with private entities. While these kinds of partnerships are often necessary and can facilitate the translation of useful technologies and practices, the commercialization of biobanks also raises a host of issues that should be considered in both the development of these partnerships and relevant policy, including:

- The potential to adversely impact the public trust;
- Consent challenges, such as the possible requirement to obtain re-consent;
- Exacerbating privacy issues and with the possible requirement for additional oversight or mechanisms to protect participants’ privacy;
- Challenges for oversight bodies, such as research ethics boards, in monitoring research;
- Possible tensions regarding the ownership and sharing of biological samples and data; and
- Uncertainty concerning the use and control of the resource if biobanks go bankrupt or lose funding support.

## Footnotes

1 While there is no general consensus on an exact definition of the term ‘biobank’, we adapt the definition provided by the Public Population Project in Genomics and Society: ‘[a]n organized collection of human biological material and associated information stored for one or more research purposes’: Public Population Project in Genomics and Society (P3G), biobank lexicon, http://www.p3g.org/biobank-lexicon; Gail E. Henderson et al., *Characterizing Biobank Organizations in the US: results from a National Survey*, 5 Genome Med. 3 (2013). As Henderson and colleagues note, biobanks come in a wide variety of forms, with significant differences in organizational and funding structures, diversity in research goals and specimen collections, and variation in partnerships with other organizations (such as universities or research centers): *Id*. The issues raised in this paper may be relevant to both general biobanks and population biobanks (a collection of biological materials that: ‘has a population basis’; ‘is established, or has been converted, to supply biological materials or data derived therefrom for multiple future research projects’; ‘contains biological materials and associated personal data which may include or be linked to genealogical, medical or lifestyle data and which may be regularly updated’; and ‘receives and supplies materials in an organized manner’): Council of Europe Committee of Ministers, Recommendation Rec(2006)4 of the Committee of Ministers to member states on research on biological materials of human origin, https://wcd.coe.int/ViewDoc.jsp?id=977859. See also, Bartha M. Knoppers et al., *Sampling Populations of Humans across the World: ELSI Issues*, 13 Annu. Rev. Genomics Hum. Genet. 395 (2012).

2 R. Jean Cadigan, *Neglected Ethical Issues in Biobank Management: results from a US Study*, 9 Life Sci. Soc. Pol. 1 (2013). See also, Henderson, *supra* note 2. It is also worth noting that Caulfield was approached by several Canadian biobank initiatives, including the province of Alberta's CBCF Tumor Bank and the Canadian Health Infant Longitudinal Development (CHILD) Study, about the issues associated with commercialization. Indeed, these communications helped to justify the production of this article.

3 Timothy Caulfield, *Commercialization Creep*, Pol. Options 20 (2012); Tania M. Bubela and Timothy Caulfield, *Role and Reality: technology Transfer at Canadian Universities*, 28 Trends Biotechnol. 447 (2010); C.J. Murdoch and Timothy Caulfield, *Commercialization, Patenting and Genomics: researcher Perspectives*, 1 Genome Med. 22 (2009); Manuel Crespo and Houssine Dridi, *Intensification of University—Industry Relationships and Its Impact on Academic Research*, 54 High Educ. 61 (2007); Francis S. Collins, *Reengineering Translational Science: the Time is Right*, 3 Sci. Transl. Med. 90cm 17 (2011); Elias A. Zerhouni et al., *The Biomarkers Consortium: public and Private Sectors Working in Partnership to Improve the Public Health*, 12 Oncologist 250 (2007); Timothy Caulfield, *Sustainability and the Balancing of the Health Care and Innovation Agendas: the Commercialization of Genetic Research*, 66 Sask. Law Rev. 629 (2003).

4 Timothy Caulfield, *Patents or Commercialization Pressure? A (Speculative) Search for the Right Target*, 22 J. Law, Information And Science 122 (2012); Genome Canada, 2012 Large-Scale Applied Research Project Competition, http://www.genomecanada.ca/en/portfolio/research/2012-competition.aspx (accessed 30 January 2014).

5 While there are, no doubt, a number of intellectual property issues relevant to this area, we will not address them in this paper (eg *Association for Molecular Pathology versus Myriad Genetics Inc.*, 133 S. Ct. 2107 (2013)).

6 Henderson, *supra* note 2; Ingeborg Meijer, Jordi Molas-Gallart and Pauline Mattsson, *Networked Research Infrastructures and their Governance: the Case of Biobanking*, 39 SPP 491 (2012).

7 Henderson, *supra* note 2; Cadigan, *supra* note 3; Aaro Tupasela and Neil Stephens, *The Boom and Bust Cycle of Biobanking—Thinking Through the Life Cycle of Biobanks*, 54.5 Croat. Med. J. 501 (2013); Mylene Deschenes et al., *Intellectual Property Rights in Publicly Funded Biobanks: much Ado about Nothing?* 29.4 Nat. Biotechnol. 319 (2011); Meijer, *supra* note 7.

8 Meijer, *supra* note 7, at 496.

9 While we do not have empirical data on the number of public biobanks seeking private partnerships, we suggest that this important question could be the subject of research in the future.

10 Henderson, *supra* note 2.

11 David B. Resnik, *Scientific Research and the Public Trust*, 17 Sci. Eng. Ethics 399 (2011); Zubin Master and David B. Resnik, *Hype and Public Trust in Science*, 19 Sci. Eng. Ethics 321 (2013). ‘Public trust’ is not a static or easily quantifiable concept. Rather, it is relational, ongoing, and changing. Additionally, as we discuss below, the ‘public’ is not a homogenous entity that speaks with one voice: there are many different groups that comprise ‘the public’, and these groups may differ in their trust of scientists. These relationships of trust may be affected by a number of different factors and change at different periods of time.

12 Michael Clemence et al., Wellcome Trust Monitor Wave 2: Tracking Public Views on Science, Biomedical Research and Science Education (London, UK 2013).

13 Id.

14 The Swinburne National Technology and Society Monitor (proposed 30 July 2012), http://www.swinburne.edu.au/lss/spru/spru-monitor.html.

15 Christine Critchley and Lyn Turney, *Understanding Australians’ Perceptions of Controversial Scientific Research*, 2 AJETS 82 (2004); Christine R. Critchley and Dianne Nicol, *Understanding the Impact of Commercialization on Public Support for Scientific Research: is It About the Funding Source or the Organization Conducting the Research*, 20 Public Underst. Sci. 347 (2011).

16 Gillian Haddow et al., *Tackling Community Concerns About Commercialisation And Genetic Research: a Modest Interdisciplinary Proposal*, 64 Soc. Sci. Med. 272 (2007); Christine R. Critchley, *Public Opinion and Trust in Scientists: The Role of the Research Context and the Perceived Motivation of Stem Cell Researchers*, 17 Public Underst. Sci. 309 (2008); Christine R. Critchley et al., *The Impact of Commercialization on Public Perceptions of Stem Cell Research: Exploring Differences Across the Use of Induced Pluripotent Cells, Human and Animal Embryos*, 9 Stem Cell Rev. 541 (2013); Alan Petersen, *Securing Our Genetic Health: Engendering Trust in UK Biobank*, 27 Sociol. Health Illn. 271 (2005).

17 Tore Nilstun and Göran Hermerén, *Human Tissue Samples and Ethics–Attitudes of the General Public in Sweden to Biobank Research*, 9 Med. Health Care Philos. 81 (2006); Timothy Caulfield et al., *Biobanking, Consent, and Control: a Survey of Albertans on Key Research Ethics Issues*, 10 Biopreserv. and Biobank. 433 (2012); Zubin Master et al., *Cancer Patient Perceptions on the Ethical and Legal Issues Related to Biobanking*, 6 BMC Med. Genomics 8 (2013); A.A. Lemke et al., *Public and Biobank Participant Attitudes Toward Genetic Research Participation and Data Sharing*, 13 Public Health Genomics 368 (2010); Susan Brown Trinidad et al., *Genomic Research and Wide Data Sharing: Views of Prospective Participants*, 12 Genet. Med. 486 (2010).

18 Caulfield et al., *supra* note 18.

19 Master et al., *supra* note 18.

20 Critchley and Nicol, *supra* note 16.

21 Lemke et al., *supra* note 18; Trinidad et al., *supra* note 18.

22 Christine Critchley, Dianne Nicol, Margaret Otlowski et al., *Public Reaction to Direct to Consumer Online Genetic Tests: Comparing Attitudes, Trust and Intentions across Commercial and Conventional Providers* (unpublished manuscript).

23 Kieran C. O’Doherty et al., *Involving Citizens in the Ethics of Biobank Research: Informing Institutional Policy Through Structured Public Deliberation*, 75 Soc. Sci. Med. 1604 (2012).

24 Dianne Nicol and Christine Critchley, *Benefit Sharing and Biobanking in Australia*, 21 Public Underst. Sci. 534 (2012).

25 Id.

26 Christine R. Critchley et al., *Predicting Intention to Biobank: a National Survey*, 22 Eur. J. Public Health 139 (2012); Nicol and Critchley, *supra* note 25; Asa Kettis-Lindblad et al., *Genetic Research and Donation of Tissue Samples to Biobanks. What Do Potential Sample Donors in the Swedish General Public Think?* 16 Eur. J. Public Health 433 (2006).

27 Herbert Gottweis et al., *Connecting the Public with Biobank Research: Reciprocity Matters*, 12 Nat. Rev. Genet. 738 (2011).

28 Critchley, *supra* note 17; Critchley et al., *supra* note 17.

29 Haddow et al., *supra* note 11. See also Alexander Morgan Capron, *Ethical Norms and the International Governance of Genetic Databases and Biobanks: Findings from an International Study*, 19 Kennedy Inst. Ethic. J. 101 (2009).

30 James P. Evans et al., *Genomics. Deflating the Genomic Bubble*, 331 Science 861 (2011).

31 Jamie Doward, *Sale of personal gene data condemned as ‘unethical and dangerous’*, The Guardian, http://www.theguardian.com/science/2013/feb/17/gene-genetic-database-nhs-genewatch (accessed 17 February 2013).

32 Timothy Caulfield, *Biobanks and Blanket Consent: The Proper Place of the Public Good and Public Perception Rationales*, 18 King's Law J. 209 (2007).

33 Lemke et al., *supra* note 18; Master et al., *supra* note 18; Trinidad et al., *supra* note 18; Caulfield et al., *supra* note 18; Zubin Master and David B Resnik, *Incorporating Exclusion Clauses in Informed Consent for Biobanking*, 22 Camb. Q. Healthc. Ethic. 203 (2013).

34 Jennifer Hochschild et al., Technology Optimism or Pessimism: How Trust in Science Shapes Policy Attitudes Toward Genomic Science (Issues in Technology Innovation, Washington, DC 2012); Michael Siegrist, *The Influence of Trust and Perceptions of Risks and Benefits on the Acceptance of Gene Technology*, 20 Risk Anal. 195 (2000); Linus Johnsson et al., *Patients’ Refusal to Consent to Storage and Use of Samples in Swedish Biobanks: cross Sectional Study*, 337 BMJ a345 (2008); Caulfield et al., *supra* note 18; Juli Murphy et al., *Public Perspectives on Informed Consent for Biobanking*, 99 Am. J. Public Health 2128 (2009); Heather L. Walmsley, *Stock Options, Tax Credits or Employment Contracts Please! The Value of Deliberative Public Disagreement About Human Tissue Donation*, 73 Soc. Sci. Med. 209 (2011).

35 Critchley and Nicol, *supra* note 16.

36 Kristin Solum Steinsbekk et al., *We’re Not in It for the Money-Lay People's Moral Intuitions on Commercial Use of ‘Their’ Biobank*, 16 Med. Health Care Philos. 151 (2013); Haddow et al., *supra* note 17.

37 Eric M. Meslin et al., *Health-Related Philanthropy: toward Understanding the Relationship between the Donation of the Body (and Its Parts) and Traditional Forms of Philanthropic Giving*, 37 NVSQ 44S (2008); Trinidad et al., *supra* note 18.

38 Existing public perception studies have focused mainly on exploring the views of the general public and patient populations respecting involvement of commercial partners in biomedical research and biobanking. This has created a gap in our understanding of the views of a range of other stakeholders including scientists, biobank personnel, public officials, regulators, and investors. Surveying the perspectives of scientists, for example, will provide knowledge about how those who conduct this research view commercialization and whether they see any ethical or practical problems arising from industry involvement. It may be, for example, that scientists who collect biobank samples anticipate ‘first right of access’ to the samples for research purposes and fear that private funding or granting access to industry researchers will create barriers.

39 Elizabeth J. Horn et al., *Engaging Research Participants and Building Trust*, 15 Genet. Test Mol. Biomarkers 839 (2011).

40 A study by Pullman and colleagues examined the value ranking made by the public when considering whether to participate in biobanking research, but did not specifically look at whether this ranking would be affected by the involvement of private, for-profit companies (Daryl Pullman et al., *Personal Privacy, Public Benefits, and Biobanks: a Conjoint Analysis of Policy Priorities and Public Perceptions*, 14 Genet. Med. 229 (2012)).

41 Zubin Master et al., *Biobanks, Consent and Claims of Consensus*, 9 Nat. Methods 885 (2012).

42 Michelle M. Mello and Leslie E. Wolf, *The Havasupai Indian Tribe Case–Lessons for Research Involving Stored Biologic Samples*, 363 N. Engl. J. Med. 204 (2010); Jay Root, *Texas officials agree to destroy babies’ blood samples after settling lawsuit*, Dallas Morning News (proposed 26 November 2010) http://www.dallasnews.com/news/state/headlines/20091223-Texas-officials-agree-to-destroy-babies-1751.ece; *Havasupai Tribe versus Arizona Board of Regents*, 204 P.3d 1063, 220 Ariz. 214 (Ct. App. 2008). See also Ricki Lewis, *Is the Havasupai Indian Case a Fairy Tale*?, Plos Blogs (proposed 15 August 2013) http://blogs.plos.org/dnascience/2013/08/15/is-the-havasupai-indian-case-a-fairy-tale.

43 Mahsa Shabani, Heidi Carmen Howard and Pascal Borry, *Commercialization of Population Biobanks: in Search of Lost Trust?* (unpublished manuscript).

44 Id.

45 Ann Silversides, *Merchant Scientists*, The Walrus, May 2008.

46 Justin E. Bekelman et al., *Scope and Impact of Financial Conflicts of Interest in Biomedical Research: a Systematic Review*, 289 JAMA 454 (2003); Joel Lexchin et al., *Pharmaceutical Industry Sponsorship and Research Outcome and Quality: systematic Review*, 326 BMJ 1167 (2003); Mohit Bhandari et al., *Association Between Industry Funding and Statistically Significant Pro-Industry Findings in Medical and Surgical Randomized Trials*, 170 CMAJ 477 (2004).

47 Bekelman et al., *supra* note 47.

48 Shabani, *supra* note 44.

49 Klaus Hoeyer, *Trading in Cold Blood?*, in Trust in Biobanking: Dealing with Ethical, Legal and Social Issues in an Emerging Field of Biotechnology 21 (2012).

50 Rebecca Skloot, *The Immortal Life of Henrietta Lacks, the Sequel*, The New York Times, (proposed 23 March 2013) http://www.nytimes.com/2013/03/24/opinion/sunday/the-immortal-life-of-henrietta-lacks-the-sequel.html; Mello and Wolf, *supra* note 43; *Havasupai, supra* note 43. Henrietta Lacks's family and the National Institutes of Health (NIH) subsequently reached an agreement which provides for the disclosure of the genomic data through a ‘controlled access’ mechanism: Kathy L. Hudson and Francis S. Collins, *Biospecimen Policy: family Matters*, 500 Nature 141 (2013). See also Timothy Caulfield and Amy L. McGuire, *Policy Uncertainty, Sequencing, and Cell Lines*, 3 Genes, Genome and Genetics (G3) 1205 (2013).

51 HUGO Ethics Committee, Statement on Human Genomic Databases (2002) http://www.hugo-international.org/img/genomic_2002.pdf; Organization for Economic Co-Operation and Development, OECD Guidelines on Human Biobanks and Genetic Research Databases (OECD Publishing 2009) http://www.oecd.org/sti/biotech/44054609.pdf; Canadian Institutes of Health Research et al., Tri-Council Policy Statement: ethical Conduct for Research Involving Humans (2010), http://www.ethics.gc.ca/pdf/eng/tcps2/TCPS_2_FINAL_Web.pdf; European Society of Human Genetics, *Data Storage and DNA Banking for Biomedical Research*, 11 Eur. J. Hum. Genet. S8 (2003).

52 John-Arne Skolbekken et al., *Not Worth the Paper It's Written on? Informed Consent and Biobank Research in a Norwegian Context*, 15 Critical Public Health 335 (2005); Béatrice Godard et al., *Community Engagement in Genetic Research: results of the First Public Consultation for the Quebec CARTaGENE Project*, 10 Community Genet. 147 (2007); Nilstun and Hermerén, *supra* note 18; Haddow et al., *supra* note 17; Kettis-Lindblad et al., *supra* note 27.

53 Caplan, Arthur L., *What No One Knows Cannot Hurt You: the Limits of Informed Consent in the Emerging World of Biobanking*, in The Ethics of Research Biobanking 25 (Springer 2009); Robert Mitchell and Catherine Waldby, *National Biobanks: clinical Labor, Risk Production, and the Creation of Biovalue*, 35 Sci. Technol. Human Values 330 (2010); Klaus Hoeyer, *The Role of Ethics in Commercial Genetic Research: notes on the Notion of Commodification*, 24 Med. Anthropol. 45 (2005).

54 Sigrid Sterckx et al., *‘Trust is Not Something You Can Reclaim Easily’: patenting in the Field of Direct-to-Consumer Genetic Testing*, 15 Genet. Med. 382 (2013); Hoeyer, *supra* note 50.

55 Under broad consent approaches, participants may give their consent to a range of future research uses of their biological material and data, though the specific nature of these future research uses is not known at the time consent is given. In contrast, specific consent mandates that researchers obtain consent for each new and distinct research project (Carlo Petrini, *‘Broad’ Consent, Exceptions to Consent and the Question of Using Biological Samples for Research Purposes Different from the Initial Collection Purpose*, 70 Soc. Sci. Med. 217 (2010)). While broad consent remains controversial in academic literature, ethical guidelines, and amongst the general public, it dominates biobank practice: Master et al., *supra* note 42. See also Clarissa Allen, Yann Joly and Palmira Granados Moreno, *Data Sharing, Biobanks and Informed Consent: a Research Paradox?*, 7 Mcgill J. L. and Health 85 (2013). Supporters of broad consent contend that the practical dilemmas associated with requiring re-consent of individual participants for new research uses of their material make broad consent necessary: Margaret F.A. Otlowski, *Tackling Legal Challenges Posed by Population Biobanks: reconceptualising Consent Requirements*, 20 Med. Law Rev. 191 (2012); Stephanie M Fullerton, *Meeting the Governance Challenges of Next-Generation Biorepository Research*, 2 Sci. Translational Med. 15cm3 (2010).

56 Additionally, commercialization raises issues related to the work of oversight bodies. For example, if a public biobank receives private funding, researchers who are using that biobank's resources may be required to obtain additional ethics approval. In other words, the involvement of a private entity may be enough to trigger the need for a reappraisal by an ethics review board. It is worth nothing that biobanks that are completely privately held (such as biobanks created by pharmaceutical companies) can go under the ethics review radar completely. This may create concerns about whether appropriate ethics oversight mechanisms are in place to deal adequately with a variety of commercialization-related concerns (eg if industry partnerships involve the use or merging of datasets).

57 Caulfield, *supra* note 28.

58 Onora O’Neill, *Some Limits of Informed Consent*, 29 J. Med. Ethics 4 (2003).

59 Kieran C. O’Doherty et al., *From Consent to Institutions: designing Adaptive Governance for Genomic Biobanks*, 73 Soc. Sci. Med. 367 (2011).

60 Bartha Maria Knoppers and Ruth Chadwick, *Human Genetic Research: Emerging Trends in Ethics*, 6 Nat. Rev. Genet. 75 (2005).

61 Trudo Lemmens and Lori Luther, Human Genetic Data Banks: From Consent to Commercialization: an Overview of Current Concerns and Conundrums (Encyclopedia of Life Support Systems, 2010), www.eolss.net.

62 Moore versus Regents of University of California, 793 P.2d 479, 51 Cal. 3d 120, 271 Cal. Rptr. 146 (1990); Greenberg versus Miami Children's Hospital Res. Inst., Inc., 264 F. Supp. 2d 1064 (S.D. Fla. 2003); Washington University versus Catalona, 490 F.3d 667 (8th Cir. 2007); National Health and Medical Research Council, Biobanks Information Paper (2010), http://www.nhmrc.gov.au/_files_nhmrc/file/your_health/egenetics/practioners/biobanks_information_paper.pdf; Timothy Caulfield and Amy L. McGuire, *Policy Uncertainty, Sequencing, and Cell Lines*, 3 G3 (Bethesda) 1205 (2013). Altao Charo, *Body of Research—Ownership and Use of Human Tissue*, 355.15 New Eng. J. Med. 1517 (2006). See also Capron, *supra* note 30.

63 Yearworth versus North Bristol NHS Trust, 2009 E.W.C.A. Civ 37 (2009).

64 Moore*, supra* note 63; Greenberg*, supra* note 63; Catalona, *supra* note 63; Caulfield and McGuire, *supra* note 63.

65 Caulfield and McGuire, *supra* note 63; Catalona, 490 F.3d 667.

66 Charo, *supra* note 63.

67 *Id.* at 1517 (‘US jurisprudence still has no coherent answer to a deceptively simple question: Do we own our own bodies?’).

68 McInerney versus MacDonald, 1992 S.C.R.2 138, 93 D.L.R. (4th) 415 (1992); Caulfield and McGuire, *supra* note 63.

69 Human Genes Research Act, RT I 2000, 104, 685, § 15.

70 Personal Genetic Information and Health Information Act, Law no. 12/2005 of 26 January, § 18.

71 Biobanks Act, No. 110/2000, § 10.

72 Caulfield et al., *supra* note 18.

73 de Faria, Paula Lobato, *Ownership Rights in Research Biobanks: do We Need a New Kind of ‘Biological Property’?* in The Ethics of Research Biobanking 263 (Springer 2009); Pilar N. Ossorio, *The Human Genome as Common Heritage: common Sense or Legal Nonsense?*, 35 J. Law Med. Ethics 425 (2007); Tania Bubela et al., *Commercialization and Collaboration: competing Policies in Publicly Funded Stem Cell Research?*, 7 Cell Stem Cell 25 (2010); Saminda Pathmasiri et al., *Intellectual Property Rights in Publicly Funded Biobanks: much Ado About Nothing?*, 29 Nat. Biotechnol. 319 (2011).

74 Jimmie Vaught et al., *Biobankonomics: developing a Sustainable Business Model Approach for the Formation of a Human Tissue Biobank*, 2011 J. Natl. Cancer Inst. Monographs 24 (2011); Martin Yuille et al., *The UK DNA Banking Network: a ‘Fair Access’ Biobank*, 11 Cell Tissue Bank 241 (2010).

75 Jocelyn Kaiser, *Human Genetics. Cash-Starved deCODE is Looking for a Rescuer for Its Biobank*, 325 Science 1054 (2009); Kevin Davies, Quebec's Genizon Biosciences Closes Its Doors (proposed 7 September 2011) http://www.bio-itworld.com/news/09/07/2011/Quebec-Genizon-Biosciences-closes-doors.html.

76 Dan Vorhaus, *Implications of Amgen/deCODE Deal for Genetic Testing Consumers*, Genomics Law Report (proposed 10 December 2012) http://www.genomicslawreport.com/index.php/2012/12/10/implications-of-amgendecode-deal-for-genetic-testing-consumers/.

77 Monya Baker, *Big Biotech Buys Iconic Genetics Firm*, 492 Nature 321 (2012).

78 Davies, *supra* note 76.

79 Génome Québec, Public Notice: genizon Biosciences Inc. (proposed 12 December 2012) http://www.genomequebec.com/81-en.news-public-notice-genizon-biosciences-inc-.html.

80 Personal communication from Catalina Lopez-Correa, CSO, Genome Quebec (proposed 21 June 2013), on file.

81 Dan Vorhaus and Lawrence Moore, *What Happens If a DTC Genomics Company Goes Belly Up?*, Genomics Law Report (proposed 18 September 2009) http://www.genomicslawreport.com/index.php/2009/09/18/what-happens-if-a-dtc-genomics-company-goes-belly-up/.

82 *Id.*; Ma'n H. Zawati et al., *Closure of Population Biobanks and Direct-to-Consumer Genetic Testing Companies*, 130 Hum. Genet. 425 (2011).

83 Cadigan, *supra* note 3, at 5–7.

84 International Cancer Genome Consortium (ICGC), International Cancer Genome Consortium (ICGC) Goals, Structure, Policies and Guidelines (proposed 8 June 2010) http://icgc.org/icgc/goals-structure-policies-guidelines; International Human Epigenome Consortium (IHEC), Goals, Structure, Policies and Guidelines (proposed 10 January 2013) http://ihec-epigenomes.org/no_cache/about/policies-and-guidelines/?cid=52&did=11&sechash=c3c24f76.

85 Yann Joly et al., *Genomic Databases Access Agreements: legal Validity and Possible Sanctions*, 130 Hum. Genet. 441 (2011). Compliance mechanisms should be in place before access is allowed, in order to ensure ethical obligations are being met.

86 Catherine Heeney et al., *Assessing the Privacy Risks of Data Sharing in Genomics*, 14 Public Health Genomics 17 (2011).

87 Dov Greenbaum et al., *Genomics and Privacy: Implications of the New Reality of Closed Data for the Field*, 7 PLoS Comput. Biol. e1002278 (2011); Yann Joly et al., *Data Sharing in the Post-Genomic World: the Experience of the International Cancer Genome Consortium (ICGC) Data Access Compliance Office (DACO)*, 8 PLoS Comput. Biol. e1002549 (2012).

88 Peter H.J. Riegman et al., *Biobanking for Better Healthcare*, 2 Mol. Oncol. 213 (2008); Kathinka Evers et al., *What Are Your Views on Commercialization of Tissues for Research?* 10 Biopreserv. and Biobank. 476 (2012).

89 Disagreements over these terms are common in contractual arrangements between industry and academia, and are not unique to the biobanking context.

90 See Capron, *supra* note 30.

91 The handling of incidental findings (IFs) has emerged as one of the most contentious issues in the area of biobanking. See, for example, S.M. Wolf, B.N. Crock, B. Van Ness, et al., ‘*Managing incidental findings and research results in genomic research involving biobanks and archived datasets*’ 14 Genet. Med. 361 (2012). We have not addressed this topic, as it is not closely related to the commercialization process. However, it should be noted that even this issue may be complicated by the involvement of industry as this invites more individuals (ie those in industry) to become aware of IFs.

92 Kristina Hug et al., *Withdrawal from Biobank Research: considerations and the Way Forward*, 8 Stem Cell Rev. 1056 (2012); Stefan Eriksson and Gert Helgesson, *Potential Harms, Anonymization, and the Right to Withdraw Consent to Biobank Research*, 13 Eur. J. Hum. Genet. 1071 (2005).

93 Timothy Caulfield et al., *Research Ethics Recommendations for Whole-Genome Research: consensus Statement*, 6 PLoS Biol. e73 (2008).

94 Critchley et al., *supra* note 17.

95 Knoppers et al., *supra* note 2. Wylie Burke et al., *Extending the Reach of Public Health Genomics: what Should Be the Agenda for Public Health in an Era of Genome-Based and ‘personalized’ Medicine?*, 12 Genet. Med. 785 (2010); Eric M. Meslin and Ibrahim Garba, *Biobanking and Public Health: is a Human Rights Approach the Tie that Binds?*, 130 Hum. Genet. 451 (2011); Stacey Periera, *Motivations and Barriers to Sharing Biological Samples: a Case Study*, 3 J. Pers. Med. 102 (2013); H3Africa | Homepage (proposed 16 August 2013) http://www.h3africa.org/.

96 Bartha M Knoppers and Rosario Isasi, *Stem Cell Banking: between Traceability and Identifiability*, 2 Genome Med. 73 (2010); Creating a Global Alliance to Enable Responsible Sharing of Genomic and Clinical Data http://oicr.on.ca/globalalliance.

